# Modulating proactive cognitive control by reward: differential anticipatory effects of performance-contingent and non-contingent rewards

**DOI:** 10.1007/s00426-018-1027-2

**Published:** 2018-05-31

**Authors:** Motonori Yamaguchi, Akio Nishimura

**Affiliations:** 1 Department of Psychology, Edge Hill University, Ormskirk, UK; 2 Department of Psychology, Yasuda Women’s University, Hiroshima, Japan

## Abstract

The present study investigated the influences of two different forms of reward presentation in modulating cognitive control. In three experiments, participants performed a flanker task for which one-third of trials were precued for a chance of obtaining a reward (reward trials). In Experiment 1, a reward was provided if participants made the correct response on reward trials, but a penalty was given if they made an incorrect response on these trials. The anticipation of this performance-contingent reward increased response speed and reduced the flanker effect, but had little influence on the sequential modulation of the flanker effect after incompatible trials. In Experiment 2, participants obtained a reward randomly on two-thirds of the precued reward trials and were given a penalty on the remaining one-third, regardless of their performance. The anticipation of this non-contingent reward had little influence on the overall response speed or flanker effect, but reduced the sequential modulation of the flanker effect after incompatible trials. Experiment 3 also used performance non-contingent rewards, but participants were randomly penalized more often than they were rewarded; non-contingent penalty had little influence on the sequential modulation of the flanker effect. None of the three experiments showed a reliable influence of the actual acquisition of rewards on task performance. These results indicate anticipatory effects of performance-contingent and non-contingent rewards on cognitive control with little evidence of aftereffects.

## Introduction

In complex operational environments such as driving a car in a heavy traffic or operating an aircraft in air turbulence, momentary distraction of attention can lead to a fatal accident. In such situations, cognitive control needs to be exercised to protect task operations from an intrusion of task-irrelevant information. A recent neurocognitive theory of cognitive control postulates two modes of cognitive control, proactive and reactive (Braver, 2012; Cohen, Botvinick, & Carter, 2000). This dual-process theory proposes that proactive control operates according to a specific goal of the task at hand; it prepares for expected changes in the environment according to the past experiences and the knowledge about relevant events by varying the emphases of different goals involved in the task as necessary. For example, the drivers become more cautious about potential hazards on a busy traffic that poses a higher risk of collision; such a precautionary state depends on proactive control that strengthens the goal of driving safely over other goals, such as reaching a destination faster. Reactive control enables a rapid response to sudden, unexpected changes in the environment, and it adjusts cognitive processes momentarily to adapt to the situation. For instance, drivers may react to a sudden appearance of a pedestrian running across a road by interrupting ongoing activities (pushing the gas pedal) and switching to an appropriate action (pushing the brake pedal); such rapid changes in the course of ongoing actions depend on reactive control.

Proactive control is characterized by sustained and anticipatory activation within the lateral prefrontal cortex (PFC), and reactive control is associated with transient activation of the PFC and other regions, such as the anterior cingulate cortex (ACC; e.g., Braver, Paxton, Locke, & Barch, 2009; Cohen et al., 2000). The ACC is thought to act as a conflict monitoring system that detects conflict in cognitive processes and sends a signal to the PFC to adjust control (Botvinick, Braver, Barch, Carter, & Cohen, 2001), but it is also involved in a range of other processes, such as sensation, emotion, memory, and attention (see, e.g., Wager et al., 2016). Importantly, studies have also noted a role of the ACC in reward-related decision making (e.g., Bush et al., 2002; Hadland, Rushworth, Gaffan, & Passingham, 2003; Takenouchi et al., 1999). Consistent with this finding, a number of studies have demonstrated links between reward-related events and these cognitive control operations (Braem, Verguts, Roggeman, & Notebaert, 2012; Fröber & Dreisbach, 2014, 2016; Hefer & Dreisbach, 2017; Locke & Braver, 2008; van Steenbergen, Band, & Hommel, 2009, 2012), but the results of these studies are not entirely consistent. In particular, some of those studies used the flanker task (Eriksen & Eriksen, 1974) and yielded contradictory outcomes of rewards on task performance (Braem et al., 2012; van Steenbergen et al., 2009; see also Dreisbach & Fischer, 2012). The present study addressed this discrepancy between studies using the flanker task, focusing particularly on the roles of anticipation and aftereffect of rewards in modulating cognitive control processes when rewards are contingent on task performance and when they are not.

### Influences of rewards on cognitive control

A number of recent studies have reported effects of reward on cognitive performance. For instance, rewards can increase response speed (Capa, Bouquet, Dreher, & Dufour, 2013; Kleinsorge & Rinkenauer, 2012; Umemoto & Holroyd, 2015). In addition, monetary incentives have shown to enhance perceptual discrimination (Engelmann, Damaraju, Padmala, & Pessoa, 2009), short-term memory (Jimura, Locke, & Braver, 2010), inhibitory control in antisaccade tasks (Chung et al., 2011; Geier, Terwilliger, Teslovich, Velanova, & Luna, 2010; Padmanabhan, Geier, Ordaz, Teslovich, & Luna, 2011), and the efficiency of switching between different tasks (Braem et al., 2012; Jiang & Xu, 2014; Nieuwenhuis & Monsell, 2002). Rewards can also influence cognitive control. Previous studies used the AX-continuous performance task (AX-CPT), a cue–probe task that distinguishes proactive and reactive control, and provided consistent results that monetary incentives increase proactive control (Fröber & Dreisbach, 2014, 2016; Hefer & Dreisbach, 2017; Locke & Braver, 2008). Others used the flanker task and showed that rewards affected sequential modulations of the flanker effect, which have been considered to reflect reactive control within the dual-process theory (Botvinick et al., 2001), but the directions of the influences differed between studies (Braem et al., 2012; van Steenbergen et al., 2009).

In the flanker task, participants are presented with a set of visual stimuli (e.g., color patches) and respond to a target while ignoring adjacent stimuli, or flankers. Flankers can be identical with the target on some trials (compatible trials) or different from the target on other trials (incompatible trials). Responses are typically faster on compatible trials than on incompatible trials, yielding the flanker effect. A robust finding in the flanker task is that the flanker effect depends on compatibility on the preceding trial, such that the effect is smaller on trials that follow an incompatible trial than on trials that follow a compatible trial (Gratton, Coles, & Donchin, 1992; Mayr, Awh, & Laurey, 2003; Torres-Quesada, Milliken, Lupiáñez, & Funes, 2014; also see Hommel, Proctor, & Vu, 2004; Stürmer et al., 2002, for similar findings in other tasks).

There are multiple mechanisms that appear responsible for this sequential modulation of the flanker effect. The dual-process theory explains this sequential modulation in terms of reactive control (Botvinick et al., 2001). According to this explanation, conflict experienced on a previous incompatible trial is registered by the ACC as an aversive signal, and this signal is sent to the PFC that increases cognitive control to resolve the conflict. Due to the increased cognitive control after a conflict trial, the compatibility effect decreases on the next trial. Consequently, the dual-process theorists have termed this phenomenon conflict adaptation effect. However, other researchers have suggested that the sequential modulation is due to priming of stimulus attributes that are presented on preceding trials (e.g., Hommel et al., 2004; Mayr et al., 2003). They have shown that responses are faster when all stimulus attributes on the preceding trial repeat (complete repetition) or all switch (complete alternation) than when some attributes repeat and others switch (partial alternation). This account does not assume resolution of conflict as a source of the sequential modulation.

In a typical flanker task, the conflict adaptation account and the priming account predict the same pattern of the flanker effects, and it appears that both mechanisms contribute to the sequential modulation (Egner, 2007). Furthermore, recent studies have suggested that a number of other mechanisms may also be involved (see Duthoo, Abrahamese, Braem, Boehler, & Notebaert, 2014). Thus, the issue of what mechanisms are responsible for the sequential modulation has been exceedingly complex, so the present study does not attempt to disentangle all of these possible accounts of the sequential modulation. Nevertheless, it is still possible to ask an empirical question of whether a certain task parameter influences the sequential modulation, apart from the underlying mechanisms that one may assume. Some studies reported that rewards reduced this sequential modulation of the flanker effect (van Steenbergen et al., 2009; van Steenbergen, Band, & Hommel, 2010), but others showed that rewards increased the sequential modulation (Braem et al., 2012; Stürmer, Nigbur, Schacht, & Sommer, 2011). To date, this discrepancy has not been addressed sufficiently.

There are a number of methodological differences between the studies that have shown the opposing effects of rewards on the sequential modulation of the flanker effect. One of the most salient differences is the way reward was provided to participants. When reward increased the sequential modulation (Braem et al., 2012; Stürmer et al., 2011), rewards were contingent on task performance and rewards were given when participants responded correctly or within a certain time window. This performance-contingent reward gives participants an incentive to perform the task better, which would increase the efficiency of proactive control operations in the PFC (Strang & Pollak, 2014). When reward reduced the sequential modulation (van Steenbergen et al., 2009), rewards were provided in a subset of trials that were chosen randomly, regardless of task performance. Such random rewards give no incentive to perform the task better, but they could influence the moods of the performer (van Steenbergen et al., 2010) or by serving as affective valence cues that influence a transient affective state (van Steenbergen, Band, Hommel, Rombouts, & Nieuwenhuis, 2015). Influences of performance-contingent and non-contingent rewards have been compared in the AX-CPT as well (Fröber & Dreisbach, 2014, 2016; also see Dreisbach & Fischer, 2012), which suggested that non-contingent rewards could increase reactive control sometimes but not always; thus, the results were not clear-cut in this respect. Therefore, although the previous studies have shown influences of rewards on cognitive control, they remain unclear as to how rewards do so. More data are needed to resolve the mixed findings.

### The present study

The present study investigated the influences of performance-contingent and non-contingent rewards on cognitive control in the flanker task. We focused on clarifying how rewards would affect the behavioral indices of cognitive control, such as response speed, the flanker effect, and its sequential modulation in three experiments. These experiments distinguished the contributions of anticipation and aftereffect of reward by examining the influences of rewards on two different types of trials. In the first type of trials, participants were precued at the beginning of a trial on which they had a chance to obtain a reward. Because participants only expected to receive a reward but had not received it yet when they performed that trial, any effects of a reward would reflect anticipation of a potential reward. In the second type of trials, participants had just received a reward (or lost a reward) on the preceding trial. Because no precue was provided on that trial, participants would not have expected a reward when they performed the trial. Any effect of a reward on these trials reflected an aftereffect of obtaining a reward.

**Fig. 1:**
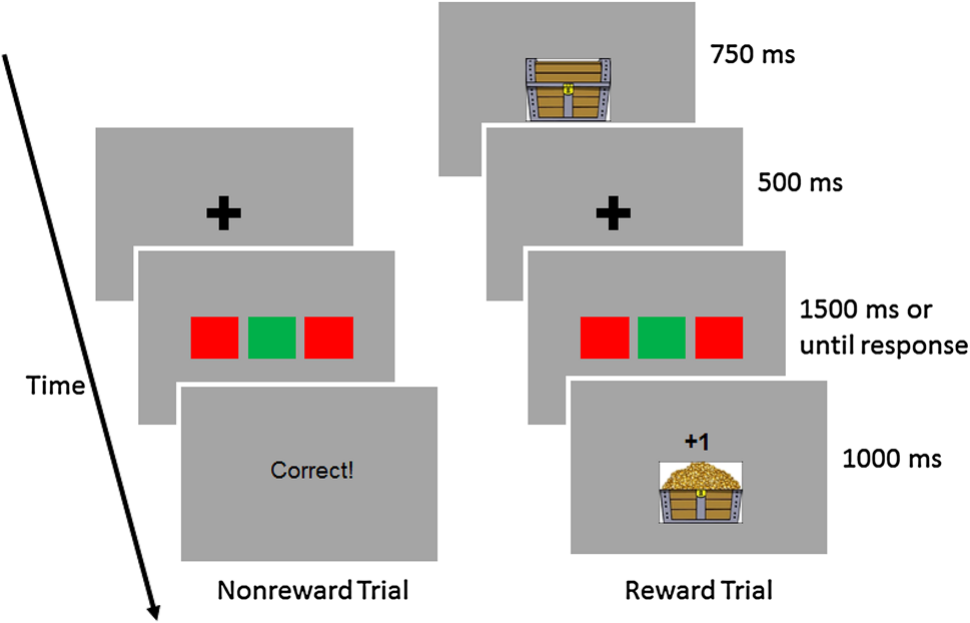
Event sequence on nonreward and reward trials

In all of the three experiments, participants responded to color targets that were accompanied by two flankers. A reward was presented on one-third of the trials, and these reward trials were precued by a visual stimulus (a drawing of a treasure box; see Fig. 1). The anticipatory effect of a reward was examined in terms of the flanker effect on the precued reward trials as compared to the flanker effect on nonreward trials that did not present any precue. The aftereffect of a reward was examined in terms of the flanker effect on nonreward trials that followed a reward trial, as compared to the flanker effect on nonreward trials that followed a nonreward trial. All three experiments used the same flanker task, but with different forms of reward presentation.

In Experiment 1, rewards were contingent on participants’ performance, such that participants gained a point (that represented the amount of a monetary reward given at the end of the session) if responses were correct, but they lost a point if responses were incorrect. In Experiment 2, rewards were independent of participants’ performance, but were randomly presented. Participants gained a point in two-thirds of the reward trials and lost a point in the remaining third. Experiment 3 was the same as Experiment 2, but the proportions of gains and losses were reversed; participants gained a point only in one-third of the reward trials and lost a point in the remaining two-third. The manipulations of the proportions of gains and losses would show the role of anticipating positive or negative outcomes. The results of the three experiments revealed differential contributions of anticipation and aftereffect of performance-contingent and non-contingent rewards in modulating cognitive control. Note that the present experiments intermixed reward and nonreward trials within the same block of trials, which addressed temporal fluctuations of cognitive control by rewards, as opposed to sustained effects that would require manipulations of rewards/penalties in separate blocks (e.g., Locke & Braver, 2008).

## Experiment 1

The aim of Experiment 1 was to examine the roles of anticipation and aftereffect of rewards in modulating the flanker effect and its sequential modulation when rewards depended on performance outcomes. Participants performed the flanker task in which they responded to the colors of target stimuli while ignoring the flankers whose colors were either identical with or different from the target color. On one-third of the trials, participants earned points if they made correct responses, and lost points if they made incorrect responses. Monetary rewards were given at the end of the session according to the accumulated points. In a similar task setting (Braem et al., 2012), the sequential modulation of the flanker effect by the preceding compatibility was found to increase on trials that followed a reward, as compared to trials that followed no reward. A similar effect of performance-contingent reward was also obtained in a different but similar task setting, namely, the Simon task, in which the sequential modulation of the Simon effect increased when good performance was rewarded as opposed to when poor performance was punished (Stürmer et al., 2011). However, these studies did not assess differential roles of anticipation and aftereffect of rewards. The present study extended these investigations by examining the role of anticipating a reward on the flanker effect by presenting a precue that signaled a forthcoming reward trial, as well as that of acquiring a reward on the preceding trial.

From the view of the dual-process theory (Braver, 2012; Cohen et al., 2000), performance-contingent rewards would serve as incentive cues that increase the efficiency of proactive control. Stronger proactive control would be exercised when a reward trial was precued, as compared to when it was not (i.e., nonreward trial). No study has examined whether anticipation of reward alone is sufficient or the actual acquisition of reward is necessary to influence the sequential modulation of the flanker effect. If the anticipation is sufficient, the flanker effect should be modulated by compatibility on the preceding trial more on reward trials than on nonreward trials. If the acquisition is necessary, the flanker effect would be affected by the preceding compatibility more when the preceding trial was a reward trial than when it was a nonreward trial. As we mentioned earlier, these predictions do not concern any specific mechanisms that may be responsible for the sequential modulation.

### Participants

Forty eight participants were recruited from the Edge Hill University community (32 females; mean age = 20.44, SD = 3.69) who received experimental credits toward their psychology module or were paid £6 for participation. They also received additional monetary rewards, which ranged from £1 to £3, depending on their task performance. All participants reported having normal or corrected-to-normal visual acuity, normal color vision, and normal hearing. The experimental protocol was approved by the Research Ethics Committee of the Psychology Department at Edge Hill University. Power analyses indicated that the current sample size would provide a statistical power of at least 0.99, assuming a medium effect size^1^ and correlation coefficient of 0.8 between within-subject measures.

### Apparatus and stimuli

The apparatus consisted of a 23-inch widescreen computer monitor and a personal computer. The experiment was controlled by E-Prime 2.0 (Psychology Software Tool, Pittsburgh, PA). Stimuli were filled squares (2.6 cm in sides) colored in green or red, which were presented against a light grey background. The fixation mark was a plus sign (“+”) printed in the 60-pt Arial font in black. The reward cue was a picture of a treasure box (see Fig. 1), and feedback on a reward trial was either a treasure box filled with a mountain of gold coins along with a fanfare sound or an empty treasure box with a buzz sound. There was no auditory stimulus along with a reward cue. Responses were registered by pressing two keys (*f* and *j*) on a standard desktop QWERTY keyboard.

### Procedure

The experiment was conducted individually under normal fluorescent lighting. Participants were seated in front of the computer monitor, wore headphones, and read instructions on the screen. They first performed 16 practice trials that consisted only of nonreward trials. Participants were then informed that some trials would be reward trials on which they could gain a point if they responded to stimuli correctly, but could lose a point if they made an error. Participants were also told that they would be paid extra monetary rewards according to the total point they earned during the session. After the instructions, participants were given another block of 20 practice trials that consisted of 6 reward trials and 14 nonreward trials, followed by four blocks of 152 test trials each (50 reward trials and 102 nonreward trials). The first and last trials of each block were always nonreward trials, and the first trial was excluded from the analysis. No repetition of reward trials was allowed; nonreward trials could repeat no more than three times in row.

The event sequences for reward and nonreward trials are depicted in Fig. 1. Each nonreward trial started with the fixation cross at the centre of screen for 500 ms, followed by a horizontal array of three filled squares. The square in the middle was the target to which participants responded, and the two adjacent squares were flankers to be ignored. The flankers were always in the same color. On a compatible trial, the target color was the same as the flanker color. On an incompatible trial, the target color was different from the flanker color. Compatible and incompatible trials occurred in an equal probability, and the target color was chosen from the two colors randomly on each trial. Participants had to respond within 1500 ms after the target onset. If the response was correct, a 1000-ms blank screen replaced the stimuli. If the response was incorrect or if there was no response within the response window, the screen was blanked for 1000 ms and a low pitch tone (400 Hz, 500 ms) was presented through the headphones within this period. There was a 500-ms blank display before the next trial started.

A reward trial was essentially the same as nonreward trials, but there was a reward cue before the fixation cross was presented. The reward cue stayed on the screen for 750 ms and was followed by the fixation cross. The target and flankers were presented in the same manner as on nonreward trials. If the response was correct, a fanfare sound was presented for 1000 ms along with the message “+ 1” and a picture of the treasure box filled with gold coins. If the response was an error, a buzz sound was presented for 1000 ms along with the message “− 1” and a picture of an empty box. A 500-ms blank display appeared before the next trial.

Response time (RT) and accuracy were recorded on each trial. RT was the interval between target onset and a depression of a response key. Responses were recorded as errors if a wrong key was pressed.

## Results

**Fig. 2:**
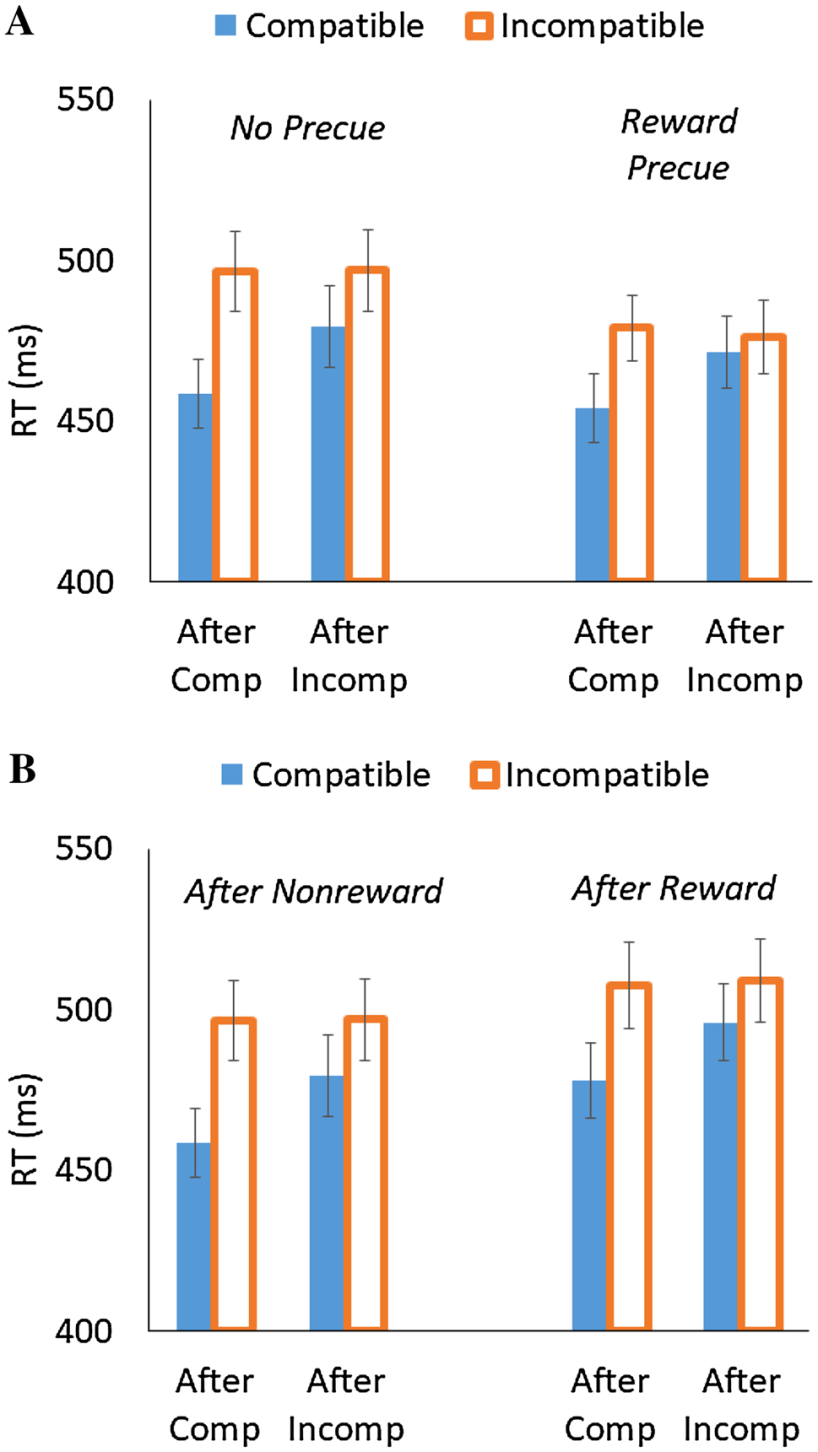
Mean response times (RT) as a function of Previous Compatibility (after compatible vs. after incompatible) and Current Compatibility (compatible vs. incompatible) in Experiment 1. **A** The role of anticipation, **B** The role of reward aftereffect

Mean RT for correct responses and percentage of error trials were computed for each trial. Trials were discarded if RT was less than 150 ms or there were no response (0.28% of all trials) or if trials followed by an error response (2.39%). RT and PE were analyzed in two ways, one that examined the role of anticipation of reward and the other that examined the role of aftereffect. RT is shown in Fig. 2, and PE is summarized in Table 1.

**Table 1:** Percentages of error trials in Experiments 1–3 (the values in the parentheses represent one standard error of the means)

		Compatible		Incompatible	
Experiment 1					
Nonreward precue	After compatible	1.08	(0.28)	2.79	(0.47)
	After incompatible	2.06	(0.39)	2.83	(0.53)
Reward precue	After compatible	1.41	(0.24)	2.37	(0.40)
	After incompatible	1.73	(0.38)	1.82	(0.35)
After nonreward	After compatible	1.08	(0.28)	2.79	(0.47)
	After incompatible	2.06	(0.39)	2.83	(0.53)
After reward	After compatible	1.66	(0.31)	1.83	(0.32)
	After incompatible	2.30	(0.39)	2.56	(0.45)
Experiment 2					
Nonreward precue	After compatible	2.44	(0.39)	7.67	(1.00)
	After incompatible	7.68	(1.00)	3.78	(0.51)
Reward precue	After compatible	7.85	(2.10)	10.39	(1.52)
	After incompatible	9.40	(1.48)	9.17	(2.06)
After nonreward	After compatible	2.62	(0.64)	7.54	(1.06)
	After incompatible	6.90	(0.92)	3.13	(0.55)
After gain	After compatible	1.36	(0.49)	9.67	(1.89)
	After incompatible	7.51	(1.09)	4.04	(1.32)
After loss	After compatible	2.44	(0.39)	7.67	(1.00)
	After incompatible	7.68	(1.00)	3.78	(0.51)
Experiment 3					
Nonreward	After compatible	2.20	(0.53)	7.97	(0.85)
	After incompatible	6.48	(0.83)	3.70	(0.62)
Reward	After compatible	4.35	(0.82)	9.35	(1.03)
	After incompatible	7.22	(1.03)	5.71	(1.13)
After nonreward	After compatible	2.20	(0.53)	7.97	(0.85)
	After incompatible	6.48	(0.83)	3.70	(0.62)
After gain	After compatible	2.39	(0.63)	8.50	(1.31)
	After incompatible	6.32	(1.00)	2.57	(0.62)
After loss	After compatible	1.33	(0.42)	8.14	(1.12)
	After incompatible	7.76	(0.94)	3.63	(0.61)

### The role of reward anticipation

To examine the role of anticipating a reward, RT and PE were computed for nonreward trials and reward trials, both followed a nonreward trial. Nonreward trials that followed a reward trial were not included in the present analysis. RT and PE were then submitted to 2 (Trial Type: reward vs. nonreward) × 2 (Previous Compatibility: after compatible vs. after incompatible) × 2 (Current Compatibility: compatible vs. incompatible) ANOVAs. All factors were within-subject variables. The results are summarized in Table 2.

**Table 2:** Results of ANOVAs on response times (RT) and percentage errors (PE) in Experiment 1

Factors	*df*	MSE	*F*	*p*	*η*_p_^2^
Reward anticipation: RT					
Trial Type (TT)	**1,47**	**2003.94**	**7.71**	**0.008**	**0.141**
Previous Compatibility (PC)	**1,47**	**502.29**	**14.65**	< **0.001**	**0.238**
Current Compatibility (CC)	**1,47**	**544.67**	**79.35**	< **0.001**	**0.628**
TT × PC	1,47	202.42	1.30	0.261	0.027
TT × CC	**1,47**	**349.18**	**11.86**	**0.001**	**0.201**
PC × CC	**1,47**	**386.28**	**25.67**	< **0.001**	**0.353**
TT × PC × CC	1,47	480.14	< 1	0.987	< 0.001
Reward anticipation: PE					
TT	1,47	6.32	1.92	0.173	0.039
PC	1,47	4.83	< 1	0.386	0.016
CC	**1,47**	**6.05**	**12.32**	**0.001**	**0.208**
TT × PC	1,47	3.89	2.41	0.127	0.049
TT × CC	1,47	5.66	2.20	0.145	0.045
PC × CC	1,47	4.91	3.95	0.053	0.078
TT × PC × CC	1,47	4.77	< 1	0.947	< 0.001
Reward aftereffect: RT					
Previous Trial Type (PTT)	**1,47**	**981.84**	**21.11**	< **0.001**	**0.310**
PC	**1,47**	**395.80**	**24.49**	< **0.001**	**0.343**
CC	**1,47**	**690.89**	**83.87**	< **0.001**	**0.641**
PTT × PC	1,47	320.57	< 1	0.844	0.001
PTT × CC	1,47	273.58	3.63	0.063	0.072
PC × CC	**1,47**	**393.82**	**21.35**	< **0.001**	**0.312**
PTT × PC × CC	1,47	416.09	< 1	0.688	0.003
Reward aftereffect: PE					
PTT	1,47	5.08	< 1	0.657	0.004
PC	**1,47**	**6.27**	**5.46**	**0.024**	**0.104**
CC	**1,47**	**7.23**	**7.10**	**0.011**	**0.131**
PTT × PC	1,47	4.50	< 1	0.685	0.004
PTT × CC	**1,47**	**4.37**	**5.71**	**0.021**	**0.108**
PC × CC	1,47	5.53	< 1	0.386	0.016
PTT × PC × CC	1,47	5.34	1.17	0.286	0.024

Bold indicates statistically significant effects at alpha = 0.05

For RT (see Fig. 2A), as indicated by the significant main effect of Previous Compatibility, responses were faster after compatible trials (*M* = 472 ms) than after incompatible trials (*M* = 481 ms) in general. In addition, the significant main effect of Current Compatibility indicated that responses were faster for compatible trials (*M* = 466 ms) than for incompatible trials (*M* = 487 ms), yielding 21 ms of the flanker effect. The flanker effect also depended on Previous Compatibility; the effect was larger after compatible trials (*M* = 31 ms) than after incompatible trials (*M* = 11 ms). Importantly, the significant main effect of Trial Type showed that responses were faster when a reward trial was cued (*M* = 470 ms) than when it was not (*M* = 483 ms), and its interaction with Current Compatibility indicated that the flanker effect was smaller when a reward trial was cued (*M* = 15 ms) than when it was not (*M* = 28 ms). These outcomes suggested that an anticipation of rewards facilitated response speed and reduced the flanker effect.

For PE, the significant main effect of Current Compatibility indicated the flanker effect of 0.88%. Although only marginal statistically, the effect tended to be larger after compatible trials (*M* = 1.33%) than after incompatible trials (*M* = 0.43%). No other effects were significant.

### The role of reward aftereffect

To examine the aftereffect of reward, the present analysis examined RT and PE for nonreward trials that followed a reward trial and those that followed a nonreward trial. They were submitted to 2 (Previous Trial Type: reward vs. nonreward) × 2 (Previous Compatibility: after compatible vs. after incompatible) × 2 (Current Compatibility: compatible vs. incompatible) ANOVAs. All factors were within-subject variables. The results are summarized in Table 2.

For RT (see Fig. 2B), the significant main effect of Previous Trial Type indicated that responses were faster on trials that followed compatible trials (*M* = 485 ms) than on trials that followed incompatible trials (*M* = 495 ms). The significant main effect of Current Compatibility also indicated that responses were faster for compatible trials (*M* = 478 ms) than for incompatible trials (*M* = 503 ms), yielding 25 ms of the flanker effect. Although statistically marginal, the flanker effect was somewhat larger after nonreward trials (*M* = 27 ms) than after reward trials (*M* = 21 ms).

For PE, the significant main effects of Previous Compatibility and of Current Compatibility showed, respectively, that responses were more accurate after compatible trials (*M* = 1.84%) than after incompatible trials (*M* = 2.44%) and that responses were more accurate for compatible trials (*M* = 1.77%) than for incompatible trials (*M* = 2.50%), yielding 0.73% of the flanker effect. The interaction between Current Compatibility and Previous Trial Type indicated that the flanker effect was larger after nonreward trials (*M* = 1.24%) than after reward trials (*M* = 0.22%). No other effects were significant.

## Discussion

The present experiment disentangled the roles of anticipation and aftereffect of performance-contingent rewards in the flanker task. Precuing a reward trial facilitated response speed and reduced the flanker effect, as compared to when a reward trial was not precued (which meant that a nonreward trial followed). Rewards facilitated RT for incompatible trials more than RT for compatible trials (19-ms facilitation for incompatible vs. 6-ms facilitation for compatible). It may be noteworthy that Wühr & Kunde, (2008) presented a precue indicating forthcoming stimulus–response compatibility in the Simon task, by which one would expect stronger proactive control, and found a larger effect of precue on compatible trials than on incompatible trials, which increased the Simon effect instead of reducing it. This finding implies that people may be able to take advantage of precued S–R compatibility more than precued S–R incompatibility, whereas the present finding implies that the anticipation of performance-contingent rewards reduced distraction from the flankers, which is consistent with the previous finding that reward enhanced the goal maintenance (Hefer & Dreisbach, 2017). The flanker effect was also reduced after reward trials (although this was significant in PE and only marginal in RT). These results may reflect a carry-over effect of increased proactive control because of a reward precue.

Nevertheless, there was little evidence that either of these effects interacted with the effect of compatibility on the preceding trial. Although the flanker effect depended on the preceding compatibility, neither the anticipation nor aftereffect of reward affected the sequential modulation. The influence of performance-contingent reward on the sequential modulation of the flanker effect was reported in a previous study (Braem et al., 2012), but the present results did not replicate the finding. However, the result are consistent with the dual-process theory that suggests that performance-contingent reward affects proactive control, not reactive control, which is also supported at least in part by recent studies (Fröber & Dreisbach, 2016; Hefer & Dreisbach, 2017), although it is not possible to point out whether the sequential modulation truly reflects reactive control in the present experiment.

Overall, the results of the present experiment suggested that anticipating performance-contingent rewards increased proactive control, reducing the flanker effect on reward trials and, to some extent, on trials that followed a reward trial. A caution has to be exercised, however, because the timing of a reward trial was slightly different from that of a nonreward trial, having an additional 750-ms precue display preceding the target. Thus, it is possible that the flanker effect was smaller on reward trails only because there was an extra time to prepare responding to the target by increasing the readiness on these trials. In addition, it may be a mere presentation of a salient precue, rather than the reward itself, that led to the difference in the flanker effect between reward and nonreward trials. If any of these factors accounted for the present outcomes, the same results should be replicated even when rewards are not contingent on performance, which was examined in Experiment 2.

## Experiment 2

In Experiment 2, rewards were provided randomly in one-third of the trials. The procedure was identical with that of Experiment 1 in other respects. On these reward trials, participants could gain or lose a point that represented monetary rewards paid at the end of the session, regardless of whether they responded correctly. Reward trials were precued in the same manner as in Experiment 1, and participants gained a point in two-third of the reward trials and lost a point in one-third of the reward trials. Although reward trials were precued, reward outcomes (gain or loss) were determined randomly without a precue, so they were unpredictable. As reward outcomes were independent of performance outcomes, there was no incentive to exert stronger proactive control even when a reward trial was precued. If the outcomes of Experiment 1 were merely due to the timing differences between reward and nonreward trials, the present experiment should replicate the same results; that is, the flanker effect should be smaller on reward trials than nonreward trials, as well as on trials that followed a reward trial than on those that followed a nonreward trial.

Alternatively, the previous studies would suggest that random rewards act as positive valence cues (Fröber & Dreisbach, 2014), which then should counteract the conflict signal from the ACC (van Steenbergen et al., 2009). If this is the case, random rewards would reduce the influence of the preceding compatibility on the flanker effect on trials that follow a reward gain, as compared to trials that follow a loss or no reward. Furthermore, to our knowledge, there has not been any study that examined the role of anticipating a random reward on proactive control and reactive control. While the previous studies showed that random rewards would affect reactive control (e.g., van Steenbergen et al., 2009), they did not examine whether the prospect of a random reward is sufficient to affect reactive control. As gains were given in two-third of reward trials and were predominant, participants could anticipate a positive reward when a reward trial was precued in the present experiment. Participants may associate the precue with a positive outcome, which then serves as an anticipatory valence cue. This should counteract the conflict signal from the preceding incompatible trial and reduce the sequential modulation of the flanker effect on reward trials. Such an outcome would suggest an anticipatory effect of non-contingent reward on reactive control.

## Method

### Participants

A new group of 48 participants were recruited from the same subject pool as in Experiment 1 (35 females; mean age = 20.44, SD = 3.27), with the same recruitment criteria.

### Apparatus, stimuli, and procedure

The apparatus and stimuli were the same as those in Experiment 1, and the procedure followed that of Experiment 1. A major modification was that gain and loss of rewards were provided randomly, irrespective of the response accuracy. After the first practice block that consisted of nonreward trials only, participants were informed that they would be presented with a treasure box on some of the trials, indicating a chance to gain a reward. They were also told that the reward would be given randomly and that it was nothing to do with their performance. Two-thirds of reward trials resulted in a gain, and one-third resulted in a loss. Participants were not informed of the proportions of gain and loss trials. The procedure followed Experiment 1 in other respects.

## Results

**Fig. 3:**
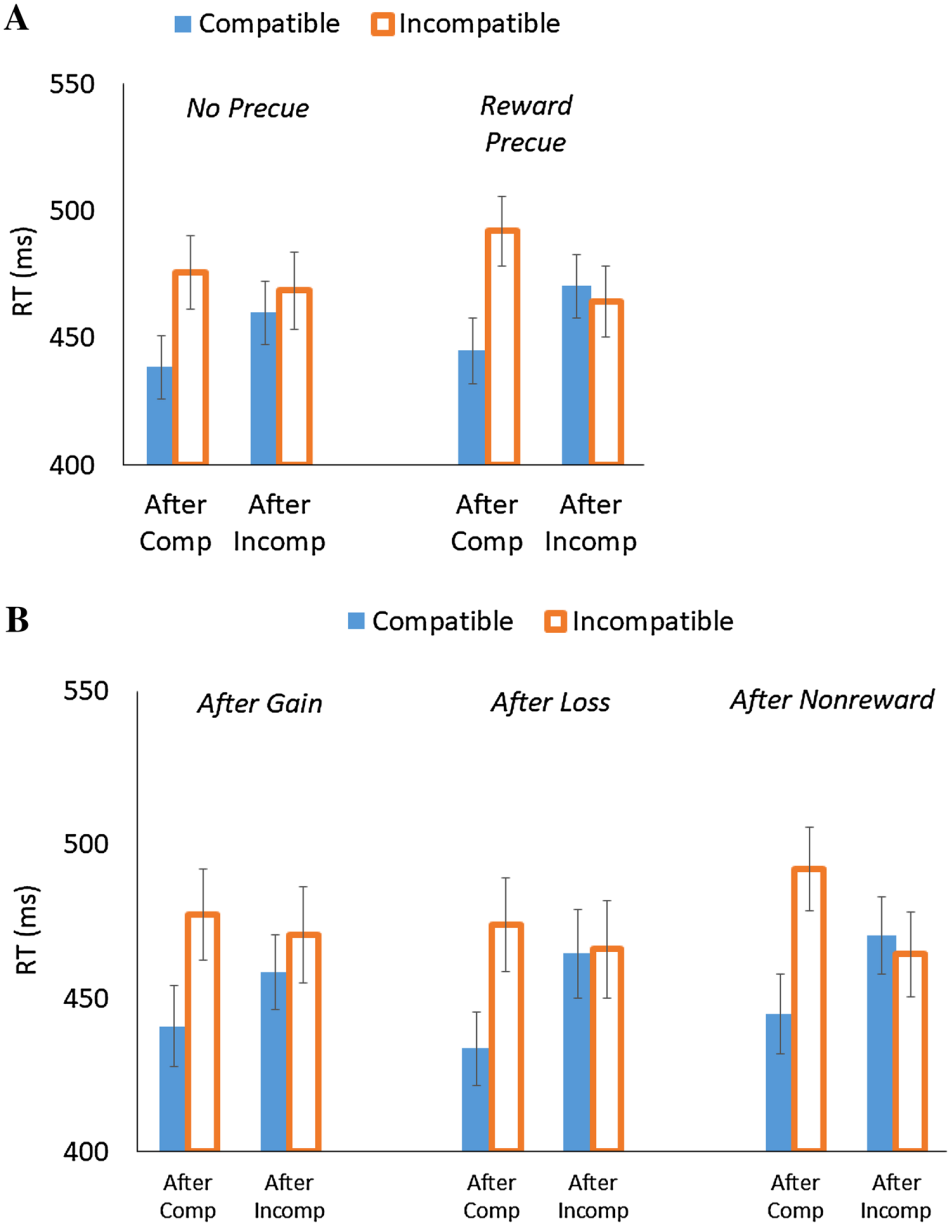
Mean response times (RT) as a function of Previous Compatibility (after compatible vs. after incompatible) and Current Compatibility (compatible vs. incompatible) in Experiment 2. **A** The role of anticipation. **B** The role of reward aftereffect

Trials were filtered in the same manner as Experiment 1(1.05% of all trials for no response or RT < 150 ms; 7.93% for trials after error). RT and PE were computed and analyzed to examine the roles of anticipation and aftereffect of random rewards separately. RT is shown in Fig. 3, and PE is summarized in Table 1.

### The role of reward anticipation

To examine the role of anticipating a random reward, RT and PE were submitted to 2 (Trial Type: reward vs. nonreward) × 2 (Previous Compatibility: after compatible vs. after incompatible) × 2 (Current Compatibility: compatible vs. incompatible) ANOVAs. Note that the analysis did not distinguish between gain and loss trials, because the reward precue only indicated that there was a potential of a reward, but not whether it was a gain or loss. As in Experiment 1, nonreward trials that followed a reward trial were not included in the present analysis. The results of ANOVAs are summarized in Table 3.

**Table 3:** Results of ANOVAs on response times (RT) and percentage errors (PE) in Experiment 2

Factors	*df*	MSE	*F*	*p*	*η*_p_^2^
Reward anticipation: RT					
Trial Type (TT)	1,47	1355.91	3.70	0.061	0.073
Previous Compatibility (PC)	1,47	596.27	1.53	0.223	0.031
Current Compatibility (CC)	**1,47**	**1381.87**	**32.86**	< **0.001**	**0.411**
TT × PC	1,47	546.16	3.15	0.083	0.063
TT × CC	1,47	676.11	< 1	0.632	0.005
PC × CC	**1,47**	**1667.69**	**24.09**	< **0.001**	**0.339**
TT × PC × CC	**1,47**	**637.34**	**5.94**	**0.019**	**0.112**
Reward anticipation: PE					
TT	**1,47**	**249.62**	**5.59**	**0.022**	**0.106**
PC	1,47	9.23	1.85	0.180	0.038
CC	**1,47**	**15.48**	**5.15**	**0.028**	**0.099**
TT × PC	1,47	11.61	< 1	0.463	0.012
TT × CC	1,47	11.58	< 1	0.483	0.011
PC × CC	**1,47**	**54.17**	**15.70**	< **0.001**	**0.250**
TT × PC × CC	**1,47**	**28.05**	**8.64**	**0.005**	**0.155**
Reward aftereffect: RT					
Previous Trial Type (PTT)	2,94	1517.33	2.44	0.093	0.049
PC	1,47	1186.93	3.45	0.070	0.068
CC	**1,47**	**2318.31**	**29.71**	< **0.001**	**0.387**
PTT × PC	2,94	1026.30	1.92	0.153	0.039
PTT × CC	2,94	941.39	< 1	0.803	0.005
PC × CC	**1,47**	**3413.34**	**15.90**	< **0.001**	**0.253**
PTT × PC × CC	2,94	905.91	2.83	0.064	0.057
Reward aftereffect: PE					
PTT	2,94	35.43	< 1	0.616	0.010
PC	1,47	23.34	< 1	0.471	0.011
CC	**1,47**	**27.04**	**7.95**	**0.007**	**0.145**
PTT × PC	2,94	25.06	< 1	0.768	0.006
PTT × CC	2,94	31.11	1.66	0.195	0.034
PC × CC	**1,47**	**74.79**	**46.88**	< **0.001**	**0.499**
PTT × PC × CC	2,94	23.72	1.43	0.246	0.029

Bold indicates statistically significant effects at alpha = 0.05

For RT (see Fig. 3A), the significant main effect of Current Compatibility indicated that responses were faster for compatible trials (*M* = 453 ms) than for incompatible trials (*M* = 475 ms), yielding a 22-ms flanker effect. This effect depended on Previous Compatibility, such that the flanker effect was 42 ms after compatible trials, but was reduced to 1 ms after incompatible trials. Importantly, these sequential modulations depended on Trial Type. When a reward trial was precued, there was a smaller sequential modulation of the flanker effect (*M* = 28 ms) than when it was a nonreward trial (*M* = 53 ms). This outcome implies that a prospect of a random reward was sufficient to reduce the sequential modulation of the flanker effect. Although only marginal, the main effect of Trial Type showed a tendency that responses are faster when a reward trial was precued (*M* = 461 ms) than when a nonreward trial was precued (*M* = 468 ms).

For PE, the significant main effect of Current Compatibility showed that responses were more accurate for compatible trials (*M* = 6.84%) than for incompatible trials (*M* = 7.75%), yielding a 0.91% flanker effect. This effect interacted with Previous Compatibility, showing that the flanker effect was 3.89% after compatible trials, but it was reversed to − 2.07% after incompatible trials. The main effect of Trial Type indicated that responses were more accurate when a nonreward trial was precued (*M* = 5.39%) than when a reward trial was precued (*M* = 9.20%), and the significant three-way interaction among Trial Type, Previous Compatibility, and Current Compatibility suggested that the sequential modulation of the flanker effect was smaller when a reward trial was precued (*M* = 2.78%) than when it was not (*M* = 9.13%).

### The role of reward aftereffect

To examine the role of reward aftereffect, RT and PE for nonreward trials were submitted to 3 (Previous Trial Type: gain vs. loss vs. nonreward) × 2 (Previous Compatibility: after compatible vs. after incompatible) × 2 (Current Compatibility: compatible vs. incompatible) ANOVAs. Table 3 summarizes the results.

For RT (see Fig. 3B), the main effect of Current Compatibility showed that responses were faster for compatible trials (*M* = 452 ms) than for incompatible trials (*M* = 474 ms), yielding a 22-ms flanker effect. Its interaction with Previous Compatibility indicated that the flanker effect was larger after compatible trials (*M* = 41 ms) than after incompatible trials (*M* = 2 ms). This reduction of the flanker effect after incompatible trials was 24 ms after a gain, 39 ms after a loss, and 53 ms after a nonreward trial, although the three-way interaction among Trial Type, Current Compatibility, and Previous Compatibility was only marginal.

For PE, the main effect of Current Compatibility showed that responses were more accurate for compatible trials (*M* = 4.75%) than for incompatible trials (*M* = 5.97%), yielding 1.22% of the flanker effect. This effect interacted with Previous Compatibility; the flanker effect was 6.16% after compatible trials, and it reversed to − 3.71% after incompatible trials. No other effects were significant.

## Discussion

The present experiment used the same procedure as in Experiment 1, except for the way rewards were provided, but the results differed markedly from those of Experiment 1. There was little influence of rewards on the overall response speed or flanker effect. This outcome is important methodologically, because it implies that the results of Experiment 1 were not due to the additional precue period that lengthened the intertrial interval of reward trials as compared to that of nonreward trials (see the General Discussion for further considerations of this issue). Hence, the present results corroborate the conclusion that the anticipation of performance-contingent rewards enhanced the response speed and reduced the flanker effect on reward trials in Experiment 1, but the anticipation of non-contingent rewards did not in Experiment 2. We also note that the overall error rate was lower in Experiment 1 (2.03%) than in the present experiment (6.33%). This may reflect the incentive for better performance in the former experiment, which was not present with random rewards in the latter.

In the meantime, the present results also showed that the reductions of the flanker effect after incompatible trials were smaller on reward trials than on nonreward trials. Such reductions were not observed in Experiment 1. Given that this occurred before the rewards were actually presented to participants, the outcomes represent an anticipatory effect of non-contingent rewards. Within the dual-process theory (Braver, 2012), this result can be interpreted that the prospect of a random reward served as a positive valence cue that counteracted the conflict signal from the preceding incompatible trial. The theory suggests that the ACC detects a conflict on incompatible trials and signals the PFC to increase cognitive control. Positive rewards can counteract this conflict signal, which then reduces reactive control, resulting in smaller reductions of the flanker effect when rewards are precued. Although only marginally significant, there were also some reductions of the sequential modulation after gain trials, as compared to those obtained after loss or nonreward trials. These outcomes are consistent with the previous study using non-contingent rewards in the flanker task (van Steenbergen et al., 2009). Interestingly, a loss trial did not lead to an increase of the sequential modulation as compared to nonreward trials. This may be because the conflict signal already had a maximum strength, so the addition of a negative event could not amplify the signal further.

The current finding of the anticipatory effect of non-contingent rewards on the sequential modulation of the flanker effect is new, and it supports the distinct roles of performance-contingent rewards in Experiment 1 and non-contingent rewards in the present experiment. We further followed up this anticipatory effect of non-contingent rewards in the next experiment. It was presumed that the reduction of the sequential modulation resulted from the predominant proportion of gain trials, which led participants to anticipate a positive reward outcome when a precue is provided. Experiment 3 reversed the proportions of gain and loss trials, so that participants should now anticipate negative outcomes more than positive ones when a reward trial is precued. We tested whether the anticipation of negative outcomes would have the same impact on cognitive control as that of anticipating positive outcomes.

## Experiment 3

The present experiment examined whether the anticipatory effect of non-contingent rewards depended on the proportion of gains and losses. In Experiment 3, two-thirds of reward trials resulted in a loss, and the remaining one-third resulted in a gain; thus, participants would anticipate more negative outcomes when reward trials were precued. If the results of Experiment 2 were due to anticipation of any non-contingent event, regardless of whether it is positive or negative, then the sequential modulation of the flanker effect should also be reduced when reward trials were precued in the present experiment, as compared to when they were not. From the view of the dual-process theory, the anticipation of negative events could amplify the aversive signal from conflict and increase reactive control. This could result in a greater modulation of the flanker effect after an incompatible trial. Nevertheless, Experiment 2 has also shown that loss trials did not lead to a greater modulation of the flanker effect, as compared to nonreward trials, suggesting that the additional aversive event did not facilitate reactive control. Therefore, the anticipation of non-contingent rewards may not influence the sequential modulation of the flanker effect when negative outcomes are predominant.

## Method

### Participants

Forty eight participants were newly recruited from the same subject pool as in Experiments 1 and 2 (29 females; mean age = 20.98, SD = 4.53), with the same recruitment criteria.

### Apparatus, stimuli, and procedure

Experiment 3 was identical with Experiment 2, except that the proportions of gain and loss trials were modified. Two-thirds of all reward trials resulted in a loss, and one-third resulted in a gain. Participants were instructed on the task in the same manner as in Experiment 2, and they were not informed of the proportions of losses and gains. Although all sessions necessarily ended with a negative overall score, as there were more loss trials, the score was inverted to a positive score after the session, so that all participants received additional compensations equivalent to those for participants in Experiment 2. Participants were not informed of this inversion until they completed the session.

## Results

**Fig. 4:**
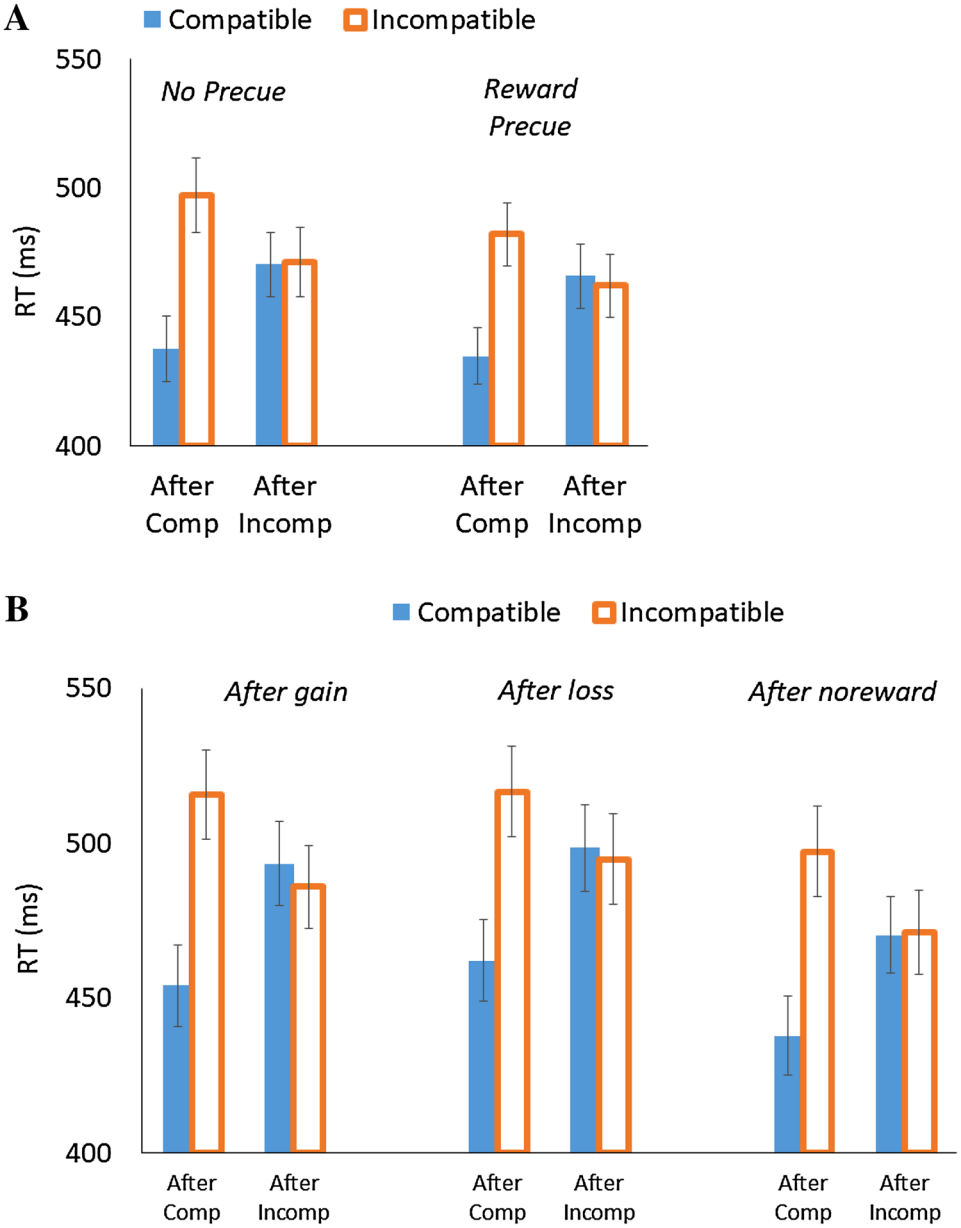
Mean response times (RT) as a function of Previous Compatibility (after compatible vs. after incompatible) and Current Compatibility (compatible vs. incompatible) in Experiment 3. **A** The role of reward anticipation. **B** The role of reward aftereffect

Trials were filtered in the same manner as in the preceding experiments (0.68% of all trials for no response or RT < 150 ms; 6.72% for trials after error). RT and PE were analyzed in the same manner as in Experiment 2. RT is shown in Fig. 4, and PE is summarized in Table 1.

### The role of reward anticipation

RT and PE were submitted to 2 (Trial Type: reward vs. nonreward) × 2 (Previous Compatibility: after compatible vs. after incompatible) × 2 (Current Compatibility: compatible vs. incompatible) ANOVAs, and the results are summarized in Table 4.

**Table 4:** Results of ANOVAs on response times (RT) and percentage errors (PE) in Experiment 3

Factors	*df*	MSE	*F*	*p*	*η*_p_^2^
Reward anticipation: RT					
Trial Type (TT)	**1,47**	**1268.20**	**4.76**	**0.034**	**0.092**
Previous Compatibility (PC)	**1,47**	**352.12**	**5.31**	**0.026**	**0.102**
Current Compatibility (CC)	**1,47**	**812.40**	**80.17**	< **0.001**	**0.630**
TT × PC	1,47	517.08	< 1	0.626	0.005
TT × CC	1,47	448.22	3.78	0.058	0.074
PC × CC	**1,47**	**863.59**	**83.53**	< **0.001**	**0.640**
TT × PC × CC	1,47	464.74	< 1	0.397	0.015
Reward anticipation: PE					
TT	**1,47**	**32.54**	**7.29**	**0.010**	**0.134**
PC	1,47	17.70	< 1	0.652	0.004
CC	**1,47**	**18.86**	**13.41**	**0.001**	**0.222**
TT × PC	1,47	17.44	< 1	0.649	0.004
TT × CC	1,47	13.54	< 1	0.740	0.002
PC × CC	**1,47**	**22.57**	**60.29**	< **0.001**	**0.562**
TT × PC × CC	1,47	13.83	1.81	0.185	0.037
Reward aftereffect: RT					
Previous Trial Type (PTT)	**2,94**	**1678.06**	**17.65**	< **0.001**	**0.273**
PC	1,47	1271.05	3.03	0.088	0.061
CC	**1,47**	**2156.39**	**50.73**	< **0.001**	**0.519**
PTT × PC	2,94	813.91	< 1	0.776	0.005
PTT × CC	2,94	830.18	< 1	0.707	0.007
PC × CC	**1,47**	**1588.16**	**87.00**	< **0.001**	**0.649**
PTT × PC × CC	2,94	973.05	< 1	0.624	0.010
Reward aftereffect: PE					
PTT	2,94	22.17	< 1	0.855	0.003
PC	1,47	24.48	< 1	0.973	< 0.001
CC	**1,47**	**29.25**	**8.85**	**0.005**	**0.158**
PTT × PC	2,94	21.33	2.17	0.120	0.044
PTT × CC	2,94	15.98	< 1	0.929	0.002
PC × CC	**1,47**	**54.87**	**62.81**	< **0.001**	**0.572**
PTT × PC × CC	2,94	21.33	< 1	0.452	0.017

Bold indicates statistically significant effects at alpha = 0.05

For RT (see Fig. 4A), the main effect of Current Compatibility indicated that responses were faster for compatible trials (*M* = 452 ms) than for incompatible trials (*M* = 478 ms), yielding a 25-ms flanker effect. The significant main effect of Previous Compatibility also indicated that responses were also faster after compatible trials (*M* = 463 ms) than after incompatible trials (*M* = 467 ms). These two factors interacted, showing that the flanker effect was 53 ms after compatible trials, and it was reduced to − 1 ms after incompatible trials. The significant main effect of Trial Type revealed that responses were faster when a reward trial was precued (*M* = 461 ms) than when it was not (*M* = 469 ms). The flanker effect was 22 ms when a reward trial was precued, and it was 30 ms when there was no precue, which was reflected in a marginally significant interaction between Trial Type and Current Compatibility. No other effects were significant.

For PE, the significant main effect of Current Compatibility indicated that responses were more accurate for compatible trials (*M* = 5.06%) than for incompatible trials (*M* = 6.68%), yielding a 1.62% flanker effect. This effect interacted with Previous Compatibility; the flanker effect was larger after compatible trials (*M* = 5.39%) than after incompatible trials (*M* = − 2.14%). The main effect of Trial Type indicated that responses were also more accurate when a nonreward trial was precued (*M* = 5.09%) than when a reward trial was precued (*M* = 6.66%). No other effects reached significance.

### The role of reward aftereffect

RT and PE for nonreward trials were submitted to 3 (Previous Trial Type: gain vs. loss vs. nonreward) × 2 (Previous Compatibility: after compatible vs. after incompatible) × 2 (Current Compatibility: compatible vs. incompatible) ANOVAs, and Table 4 summarizes the results.

For RT (see Fig. 4B), the main effect of Current Compatibility indicated that responses were faster for compatible trials (*M* = 469 ms) than for incompatible trials (*M* = 497 ms), yielding a 28-ms flanker effect, and its interaction with Previous Compatibility indicated that the flanker effect was larger after compatible trials (*M* = 58 ms) than after incompatible trials (*M* = − 3 ms). The main effect of the Previous Trial Type indicated that responses were fastest when the previous trial was a nonreward trial (*M* = 469 ms), intermediate when it was a gain (*M* = 487 ms), and slowest when it was a loss (*M* = 493 ms). No other effects were significant.

For PE, the main effect of Current Compatibility indicated that responses were more accurate for compatible trials (*M* = 4.41%) than for incompatible trials (*M* = 5.75%), yielding a 1.34% flanker effect, and its interaction with Previous Compatibility showed that the flanker effect was larger after compatible trials (*M* = 6.23%) than after incompatible trials (*M* = − 3.55%). No other effects were significant. The overall error rate was (*M* = 5.47%), similar to Experiment 2, but still lower than Experiment 1; as suggested earlier, the lower error rate in Experiment 1 likely reflected the performance-contingent incentives.

## Discussion

The present experiment examined whether the anticipatory effect of non-contingent rewards depended on the proportions of reward outcomes. In contrast to the results of Experiment 2, which showed that the anticipation of non-contingent rewards reduced the sequential modulation of the flanker effect when gains were predominant, the present results revealed little influence on the sequential modulation when loss trials were predominant. These results support the conclusion that the anticipation of a positive outcome drives the influence of non-contingent rewards, not just a possibility of any non-contingent event. Again, these results also indicated that any anticipatory effects observed in the preceding experiments were not explained by the timing of the stimulus events alone (i.e., the additional 750 ms for the reward precue on reward trials), because the only difference between Experiment 2 and Experiment 3 was the proportion of gains and losses. Therefore, the contents of the events that followed the reward precues did matter.

Two outcomes of the experiment were not predicted a priori, so they require some speculations. The first unexpected outcome was that the anticipation of a reward trial facilitated response speed and reduced the flanker effect. These observations were similar to those obtained with performance-contingent rewards in Experiment 1, and they indicated that stronger proactive control was exerted when a reward trial was precued. An anecdotal explanation of these findings would be that some participants desperately tried to figure out why they were being punished and attempted to perform the task better to avoid losses, despite the fact that they had been informed that reward was determined randomly. These participants might have exercised stronger proactive control on a reward trial. The present results also suggested that responses were particularly slowed after a loss trial, which was not observed when gain trials were predominant in Experiment 2. Such slowing might have occurred if participants tried to figure out an explanation for the loss after the trial. Our speculation of guessing about random events may be akin to recent findings that cognitive control increased when participants guessed upcoming tasks that were generated randomly but not when they ‘chose’ upcoming tasks that might be accepted or denied (Kleinsorge & Scheil, 2016, 2018).

The second unexpected outcome was that there was little influence on the sequential modulation of the flanker effect after a gain trial. If a gain served as a momentary positive valence cue that counteracted the conflict signal, the sequential modulation should have been reduced after a gain (van Steenbergen et al., 2015). A possible reason for not having observed a reduction of the sequential modulation in the present experiment is that negative moods had developed when participants experienced more losses, and these negative moods might have overridden and suppressed the influence of gains; that is, participants were no longer pleased with momentary gains, as they were always losing overall. In a previous study, the sequential modulation has been shown to depend not only on reward-related factors, but also on the moods of participants (van Steenbergen et al., 2010). With the predominance of losses in the present experiment, many participants expressed frustration during debriefing at the end of the session. Thus, participants were in negative moods while performing the task, so a momentary positive valence cue might not have been as effective as when participants were in positive or neutral moods. This would explain the lack of influence of positive reward on the sequential modulation of the flanker effect.

Despite these unexpected (but reasonable in retrospect) outcomes, the present experiment supported the claim that the anticipatory effect of non-contingent reward on the sequential modulation of the flanker effect depended on the predominance of the outcomes. Therefore, the findings in Experiment 2 are not due to extraneous factors (e.g., the timing of the stimulus presentations) other than the reward anticipation and aftereffect. The results indicated that there are differential influences of performance-contingent and non-contingent rewards on proactive and reactive controls.

## General discussion

A popular neurocognitive theory of cognitive control distinguishes proactive and reactive controls as the sources of behavior regulation (e.g., Botvinick, 2007; Braver, 2012). This dual-process theory is well grounded in behavioral and cognitive neuroscience evidences, and there is a growing interest as to how these cognitive operations interplay with motivational and emotional factors (e.g., Botvinick & Braver, 2010; Braem et al., 2012; Fröber & Dreisbach, 2016; Hadland et al., 2003; Stürmer et al., 2011; van Steenbergen et al., 2010). The present study aimed at distinguishing the roles of anticipation and aftereffect of two forms of reward presentation in proactive and reactive controls. In the three experiments, the contingency of reward on task performance was manipulated. In Experiment 1, reward was contingent on task performance, where participants gained a reward when they performed the task correctly, but they lost it when they made an error. Precuing a reward trial facilitated the overall response speed and decreased the flanker effect, which suggests an increase in proactive control, consistent with the previous findings that incentives enhance attentional control and reduce conflict (Engelmann et al., 2009; Padmala & Pessoa, 2011). On the other hand, Experiment 1 offered little evidence that performance-contingent rewards influenced the sequential modulation of the flanker effect. This outcome contradicts a previous study that reported an increased sequential modulation after a performance-contingent reward was provided (Braem et al., 2012), but it is consistent with recent studies using the AX-CPT that suggested that performance-contingent rewards increased proactive control but had little influence on reactive control (Fröber & Dreisbach, 2014; Hefer & Dreisbach, 2017).

With non-contingent rewards, the anticipation of a positive reward was shown to reduce the sequential modulation of the flanker effect in Experiment 2, but the anticipation of a negative reward had little impact on the sequential modulation in Experiment 3. The previous studies have shown that there were smaller sequential modulations of the flanker effect after a non-contingent reward was acquired (van Steenbergen et al., 2009, 2012), which is consistent with the results of Experiment 2. However, these studies have not separated the anticipatory effect of non-contingent rewards from their aftereffects. Because the three experiments of the present study used the same procedure except for the contingency of rewards, the discrepancies of the results point to the influence of the contingency, not other peripheral factors such as timing of trial events that were slightly different between reward and nonreward trials. Instead, the present findings provide a novel conclusion that, based on the dual-process theory, the prospect of a non-contingent reward is sufficient to modulate reactive control.

According to the dual-process theory, reactive control depends on aversive conflict signals from the ACC to the PFC, and a positive valence cue can cancel out the aversive signals, resulting in a reduction of the sequential modulation of the flanker effect. Within this framework, the present results imply that anticipation of a positive reward serves as a positive valence cue and is sufficient to cancel the aversive signal. A previous study also has shown that the sequential modulation is reduced under positive moods (van Steenbergen et al., 2010), which suggests a sustained effect of positive affect. As the present study varied positive and negative rewards across trials in a random fashion and the effects of rewards were examined by comparing trials within the same block, the findings reflected transient effects of non-contingent rewards on reactive control. Thus, together with van Steenbergen et al.’s finding, the present study add that there are both sustained and transient effects of non-contingent rewards on reactive control, which can be contrasted to the previous findings that there are both sustained and transient effects of performance-contingent rewards on proactive control (e.g., Chiew & Braver, 2013; Engelmann et al., 2009; Locke & Braver, 2008).

Interestingly, there was little influence of anticipating negative rewards on reactive control. When losses were predominant, the transient effect of positive rewards was also absent. A possible reason for this outcome is that the anticipation of negative rewards led to negative moods, which thus produced a sustained effect of negative rewards and suppressed the transient effect of positive rewards. It is equally possible that the influence of reward aftereffect is contingent on the anticipation of rewards, such that positive rewards can affect proactive or reactive control only when positive rewards have been anticipated prior to the reward outcome (cf. Notebaert & Braem, 2015). It may be the consistency between expected and actual outcomes, not mere positive valence cue that counteracted the conflict signal on incompatible trials and decreased reactive control on the following trial. This possibility needs to be scrutinized in future investigations.

A possible limitation of the present study was mentioned in Experiment 1 and addressed in Experiments 2 and 3, which was that any differences between reward and nonreward trials in the current procedure could reflect the timing differences between the two types of trial. Throughout the three experiments, a reward trial was preceded by a reward cue that appeared for 750 ms, which did not appear on a nonreward trial. In a study that used a Stroop-like task involving a categorization of facial stimuli into male or female that were accompanied by the word MALE or FEMALE, the sequential modulation of the Stroop-like effect decreased as the intertrial interval (ITI) increased (Egner, Ely, & Grinband, 2010). If this time course of the sequential modulation is responsible solely for the present findings, one would have to speculate that the lack of the influence of performance-contingent reward in Experiment 1 reflected a decay of the sequential modulation, whereas the presence of the influence of performance non-contingent reward in Experiment 2 reflected a facilitation. Furthermore, the lack of the influence of performance non-contingent reward in Experiment 3 should also reflect a decay, that is, in the opposite direction to that obtained in Experiment 2. Consequently, the time course of the sequential modulation should have depended on the contingency of reward on performance as well as the predominance of gains or losses of performance non-contingent reward. This alternative interpretation is highly speculative and opportunistic, and we also note that it only differs from our conclusion as to whether reward affects reactive control indirectly via intertrial interval or directly via anticipation of reward. Moreover, the alternative account does not have much to say about why proactive control also reduced when performance-contingent reward was precued (Egner et al. did not report any effect of ITI on overall RT or Stroop-like effect), which leaves us flat as to how it explains the present results. Nevertheless, our conclusion that the performance contingency and the predominance of performance non-contingent reward gains play important roles in cognitive control still remains intact even if the ITI effect depends on the type of reward and the predominance of gain/loss. Considering the fact that there are several possible mechanisms that could give rise to the sequential modulation (Botvinick et al., 2010; Duthoo et al., 2014; Hommel et al., 2004), one cannot conclude based on the present results how the two forms of reward presentation influence reactive control, but it seems safe to conclude that performance-contingent rewards influence proactive control, whereas non-contingent rewards influence reactive control directly or indirectly.

The conclusion is generally consistent with the previous findings using the AX-CPT (Fröber & Dreisbach, 2014; Hefer & Dreisbach, 2017). The results further suggest that the anticipation of rewards plays an important role in both forms of reward presentation, although they are expressed differently. It would be interesting to examine in future studies as to whether anticipation is a prerequisite to observe modulations of proactive and reactive control in the two forms of reward presentation. Researchers have suggested that rewards have both motivational and affective components (Berridge & Robinson, 2003), and these components may have different influences on cognitive control (Chiew & Braver, 2011). Another study by Braem et al. (2013) found in a task-switching situation that performance-contingent presentation of positive valenced pictures influenced task-switching cost differently from performance non-contingent presentation of positive valenced pictures, consistent with the present findings. It has further been suggested that the affective component of reward depends on the actual delivery of a reward (Notebaert & Braem, 2015), but the present study indicates that anticipation of a reward is sufficient for the affective component to modulate reactive control without actual delivery. It is to be seen whether anticipation is also sufficient for purely affective cues, such as pleasant vs. unpleasant pictures, to influence cognitive control (e.g., Dreisbach & Goschke, 2004; Kuhl & Kazén, 1999; Phillips, Bull, Adams, & Fraser, 2002; Rowe, Hirsh, & Anderson, 2007; Van der Stigchel, Imants, & Ridderinkhof, 2011). Provided that the influences of affective stimuli or moods on cognitive processes still remain unclear in other domains as well (e.g., Bruyneel et al., 2013), such investigations would provide better understanding of the important interplay between cognitive control and affective and motivational processes.

## Footnotes

1 van Steenbergen et al. (2009) had reported a Cohen’s *d* of 0.49 (medium effect size) for the effect of reward on the sequential modulation. Although Braem et al. (2012) and Stürmer et al. (2011) did not report effect sizes, the range of their sample was 21–44 participants.
